# Feeling all alone in the world – experiences of patients with a neurological disease during a COVID‐19 visitor ban: An interview study

**DOI:** 10.1002/nop2.1278

**Published:** 2022-06-21

**Authors:** Mia Ingerslev Loft, Ingrid Poulsen, Rikke Guldager

**Affiliations:** ^1^ Department of Neurology Rigshospitalet Copenhagen Denmark; ^2^ Health, Department of Public Health, Research Unit for Nursing and Healthcare Aarhus University Aarhus Denmark; ^3^ Department of Neurorehabilitation, TBI Unit Rigshospitalet Hvidovre Denmark; ^4^ Neurosurgical Department Rigshospitalet Copenhagen Denmark

**Keywords:** interviews, neurology, relative

## Abstract

**Aim:**

The aim of this study was to explore how patients with neurological disease experienced a COVID‐19 visitor ban and to identify ways of improving the quality of care.

**Background:**

In March 2020, a temporary visitor ban was introduced in Danish hospitals to reduce the spread of COVID‐19. This led to changes in clinical practice, leaving patients without their loved ones beside them. Since neurological patients are already considered vulnerable due to physical, and sometimes cognitive impairment, we urgently wished to investigate these circumstances to facilitate appropriate support.

**Design:**

This study was conducted using a qualitative explorative design.

**Methods:**

Fourteen patients with neurological disease were interviewed using a semi‐structured interview guide. Data were analysed through inductive thematic analysis.

**Results:**

For most patients, being hospitalized during the COVID‐19 visitor ban was a painful experience with the potential to negatively influence both their mental and physical health.

## 1 INTRODUCTION

On 18 March 2020, a temporary visitor ban was introduced in Danish hospitals to reduce the spread of the COVID‐19 virus. This ban meant restrictions on the physical presence of relatives to hospitalized patients. Exceptions were made for maternity wards, paediatric patients, patients in need of end‐of‐life care and patients suffering from severe neurocognitive disability (STPS, 2020). The COVID‐19 visitor ban led to sudden changes in clinical practice, leaving neurological patients without their loved ones in potentially life‐threatening situations or throughout long and lonely hospitalizations.

So far, evidence on how COVID‐19 restrictions have affected our lives is still sparse. It is, however, clear that the pandemic has had a severe impact on many aspects of our everyday lives (Day et al., 2020; Keller et al., 2020; UN, 2020) by influencing our general well‐being and our physical, mental, psychosocial and economic health (WHO, 2020). Patients who are already vulnerable will presumably be at risk of further marginalization. Thus, it seemed important to explore these patients' needs and focus on how to give appropriate support throughout the pandemic and in the future (Day et al., 2020; Galea et al., 2020).

## 2 BACKGROUND

Despite therapeutic advances, neurologic disorders are still considered severe due to the high associated mortality rate (Wiśniewski, 2020) and the risk of repercussions in the form of physical, cognitive, communicative, behavioural and psychosocial difficulties (Ates et al., 2018; Hesselvig et al., 2020).

As medical advances accelerate, increasing numbers of neurological patients are fortunate enough to survive critical illness. The survivors, however, often suffer new or worsened physical, cognitive and emotional sequelae, which increases the risk of morbidity, mortality and reduced quality of life (LaBuzetta et al., 2019). Some neurological patients will need long‐term care following hospitalization, but finding appropriate disability support and health and rehabilitation services is often challenging. Only integrated care can address such multiple needs (Foster et al., 2015).

Because of the severity and complexity of neurological disorders, relatives have traditionally – until the emergence of COVID‐19 – played a statistically significant role during hospitalization (Guldager et al., 2019). Having relatives around has previously been seen as essential support that helps neurological patients maintain hope and motivation. Furthermore, relatives play several important roles by participating in nursing care, ward rounds and meetings with nurses. Patients claim that they need to talk over their situations with their loved ones. Some patients also need their assistance to remember and keep track of the extensive information their hospitalization can require (Loft et al., 2019).

However, even with the relatives present, a recent study illuminated that patients hospitalized for rehabilitation after a stroke struggled with existential thoughts and concerns while longing for human contact and support from nurses (Loft et al., 2019). This was in line with another study showing that older neurological patients generally scored low on life satisfaction (Wysokiński et al., 2018). Given that emotional distress during hospitalization is the best predictor of future emotional distress (LaBuzetta et al., 2019), it is worth considering how best to deliver adequate support for these patients.

Amy Freeman‐Sanderson et al. (2020) argued that the effort to reduce transmission of the COVID‐19 virus not only keeps patients and their loved ones apart but simultaneously minimizes and disrupts in‐hospital communication and interaction between patients and nurses. Facial expressions, gestures and voice qualities are disrupted by the needed protective equipment, such as masks, face shields, goggles and gowns (Freeman‐Sanderson et al., 2020). Since communicative impairments are already a common challenge for neurological patients, these obstacles might further exacerbate their conditions leading to a need to address this issue (O'Halloran et al., 2009; O'Halloran et al., 2012). Additional investigations seem necessary to fully understand the complex healthcare needs of neurological patients under COVID‐19 restrictions.

We need to rapidly increase our knowledge in this area to meet the healthcare needs of patients prompted by the pandemic. This study aimed to explore how neurological patients experienced the visitor ban and hopefully identify ways to improve care quality.

## 3 METHODS

### 3.1 Design

A qualitative explorative design was chosen for this study, and 14 semi‐structured interviews with neurological patients were conducted, followed by a thematic analysis.

### 3.2 Participants

The included participants were recruited using purposive sampling. To ensure maximum variation in age, gender, neurological disease and disease severity, participants representing patients admitted for short courses of treatment (e.g. in neurosurgical departments for only a few days) and patients admitted for rehabilitation of up to several months were invited. All 14 invited patients accepted the invitation to participate. For an overview of the included patients, see Table 1.

**TABLE 1:** Patients' characteristics

Participant *n*	Age	Admission diagnosis	Department	Gender
1	80	Stroke	Stroke Rehabilitation Unit	Female
2	81	Stroke	Stroke Rehabilitation Unit	Female
3	61	Cancer/paraneoplastic polyneuropathy	Acute Neurological Unit	Female
4	68	Awaiting diagnosis	Acute Neurological Unit	Female
5	56	Awaiting diagnosis	Acute Neurological Unit	Male
6	54	Shunt dysfunction after stroke	Neurosurgical Unit	Male
7	55	Aneurism	Neurosurgical Unit	Female
8	42	Meningeal tumour	Neurosurgical Unit	Female
9	44	Subarachnoid haemorrhage	Semi Intensive Care Unit	Female
10	48	Aneurism	Neurosurgical Unit	Male
11	49	Subarachnoid haemorrhage	TBI rehabilitation Unit	Female
12	62	Stroke	TBI rehabilitation Unit	Male
13	59	Meningeal tumour	TBI rehabilitation Unit	Male
14	68	Intracerebral haemorrhage	TBI rehabilitation Unit	Male

### 3.3 Setting

This investigation was conducted in three different neurological facilities in a Danish University Hospital: (a) a neurological department consisting of an acute stroke clinic, two in‐hospital stroke rehabilitation units, four acute neurology units and several specialized outpatients clinics (e.g. for headache disorders, dementia, multiple sclerosis); (b) a neurosurgical department consisting of a 33‐bedward and a semi‐intensive care unit with 10 beds; and (c) a 22‐bed highly specialized rehabilitation department for patients with severe traumatic brain injury.

### 3.4 Data collection

A semi‐structured interview guide was constructed. A prompting technique was used, and the interview guide was only loosely followed to create a better conversational flow and help participants feel more relaxed. All 14 interviews conducted were recorded and transcribed verbatim by the author team, all experienced with doing qualitative research.

### 3.5 Data analysis

Influenced by Braun and Clarke (2006), the data were analysed through computer‐assisted inductive thematic analysis using QSR International NVivo version 10. Thematic analysis is a suitable method for identifying, analysing and reporting themes within data (Braun & Clarke, 2006).

Braun and Clarke described the following six steps: (a) familiarizing yourself with your data, (b) generating initial codes, (c) searching for themes, (d) reviewing the themes, (e) defining and naming the themes and (f) producing a report (Braun & Clarke, 2006).

The transcribed data were read thoroughly by all the authors. All meaningful segments of text were labelled using descriptive codes. Codes describing similar contents were grouped and, after that, reviewed and, if necessary, recoded. Reduced data from all groups were then collated to identify emerging themes. All themes were reassessed to ensure the internal consistency and validity of the overall data. The reassessment resulted in some of the themes being subdivided or renamed to reflect their content more precisely.

The methodological rigour of this study was established by following Lincoln and Guba's (1985) suggestions for ensuring trustworthiness through four quality criteria: dependability, credibility, confirmability and transferability. The solid research design ensured the dependability of the data collection and analysis. The entire research group was involved throughout the process and participated in developing the research questions, creating the interview guide, reviewing the interview transcripts, and analysing and discussing the findings. To ensure credibility, the data were shared within the research group. The analytical process continued until the team reached an agreement on themes and findings. Confirmability was confirmed by including different patients representing various lived experiences. Finally, transferability was ensured by providing contextual data and information about the included patients and the study's setting. The Consolidated Criteria for Reporting Qualitative Research (COREQ) was followed (Supplementary File S1).

### 3.6 Ethical considerations

Approval from the Danish Data Protection Agency was obtained, and the study was conducted according to the principles of the Declaration of Helsinki. Written and verbal information was given to participants before obtaining their informed consent to participate in the study. Participants were informed of the study aim, and voluntary nature of the research and that withdrawal from the study was possible at any time with no implications for future treatment or rehabilitation. Research within the neurological field requires increased attention to the patient's cognitive state and whether they can understand the meaning of the consent, inclusion and acceptance of entering into an interview. Hence, before inclusion, healthcare professionals from the patient's unit assessed the individual patient's cognitive state to ensure it was ethically sound to include the patient. The interviews were conducted respectfully, considering the patient's condition, while keeping attention on the potential emotional distress the interview could unlock. If a patient became distressed during the interview, we tried to support the patent emotionally and asked permission to inform their primary nurse, enabling follow‐up and further emotional support. According to the data protection regulations, data was stored in a closed folder, and all participants’ names were changed to ensure anonymity.

## 4 RESULTS

Based on the analysis of the 14 interviewed patients' experiences, we constructed four themes: *torn between societal sacrifices and meeting one's own needs, feeling all alone in the world, ways of overcoming the visitor ban, and the importance of compassionate nurses*. Each theme captured important experiences related to our research question, helping us uncover how patients with neurological diseases experienced the visitor ban.

### 4.1 Torn between societal sacrifices and meeting one's own needs

How the visitor ban was perceived differed considerably between patients. An overall impression, however, was that the visitor ban seemed to leave the patients feeling torn. On the one hand, they acknowledged the need for a COVID‐19 visitor ban to limit the spread of the virus in society; on the other hand, this caused them great suffering, as it led to unmet emotional needs due to not having their loved ones beside them. They faced a tough battle between sacrificing for the good of society and suffering emotional deprivation during a difficult period in their lives. It seemed that the experience was associated with the length of the hospitalization and the severity of the neurological illness since some patients on the neurosurgical ward characterized by short hospitalizations said that the visitor ban also had benefits:

*It's really cruel to say, but I actually think it's been very nice. And it's not because I do not want my parents, my boyfriend, and my girlfriends to visit, but it's just nice being able to give up this responsibility*. *(patient 8)*

Another patient responded to the question, “So you have not missed having relatives visit?” with “No, but I have also slept most of the time. Had I been rested and awake, I might have found it a bit boring” (patient 9).

The visitor ban thus gave some patients peace of mind and relieved them of the responsibility for deciding whether they wanted relatives to visit. It gave them much‐needed time to recover, for example, after an operation, and some described this as a relief.

However, most patients were considerably emotionally affected by the visitor ban:

R *As I hear it, the fact that you have not been able to have your relatives visit you causes distress?*

P *Yes, yes. They should preferably be here*.

R *Are there any benefits of having no visitors?*

P *NO (speaks for the first time in a clear voice). No, I don't think so. If only they could visit me one at a time, I would be happy. (patient 2)*.

Most patients had not been informed about the visitor ban by healthcare professionals. Some had received written material but did not have the strength to read it. Many had, nevertheless, heard of the ban before their admissions through the media or other channels. However, it had come as a surprise for some when they had been picked up by their patient transportation service or when they arrived at the hospital in an emergency. The fact that they were not allowed to have their relatives with them had been a painful experience. All interviewed patients were aware that a visitor ban existed, and nearly all were aware that the purpose was to reduce the risk of spreading the infection.

Despite their emotional struggles, the patients all accepted the ban and came to terms with it. It was considered an acceptable and understandable safeguard for society and themselves, even though they would have liked it to be otherwise. It seemed important to them that the ban affected everyone. However, it made it harder to accept if the rules were bent and a fellow patient, for instance, was allowed visits or was able to meet their relatives outside the ward.

### 4.2 Feeling all alone in the world

Despite their overall understanding of the need for a COVID‐19 visitor ban, the patients described that the ban had consequences and disadvantages for both them and their relatives. Patients who had previously had their relatives around described their presence as supportive and able to give peace of mind, whereas lacking their presence could make them feel all alone in the world. Not having their loved ones present and participating in the hospitalization was difficult to bear. Some patients further described how the lack of contact with their relatives directly influenced their moods; they easily became upset and said that they had lost their motivation to get better. They also described that the situation affected their desire to exercise, eat and drink:

*I feel even less like eating and drinking. Then it just doesn't matter at all (the voice breaks, and the patient starts crying)* *(patient 3)*

Not having relatives around could feel like a statistically significant loss, which caused sadness and irritation for some. Some patients also said that the days became too long and monotonous, with no positive interruptions or nothing to look forward to.

They also described that the distance affected their relatives because they were not able to see the patients and gain insight into their conditions and development of the disease:

*He was very surprised because, back then, I could walk a little and was capable of doing some things. Suddenly, I couldn't use either my arms or legs, so it was a huge, huge shock to him. And he couldn't understand it, you know. He was not able to follow the process when normally he would have been there and able to see…* *(patient 3)*

The distance could reduce the relatives' involvement and give both relatives and patients extra worries because they could not see how they were doing with their own eyes.

The hardest part, however, was not having someone to share the negative thoughts and difficult situations with, having to manage without relatives as an extra pair of ears and not having them physically present to give comfort, hope and affectionate gestures such as hugs or hand‐holding:

*It is hard that you cannot see your relative or have a hug when you need it. That's the hardest thing, right? Of course, my husband had a really tough time, in the beginning, realising how fast my condition was worsening. He cannot make sense of it, so he goes around and speculates…* *(patient 4)*

Having relatives around for most patients was described as giving peace or providing support since otherwise, there was a chance of feeling all alone in the world.

### 4.3 Ways to overcome the visitor ban

The COVID‐19 visitor ban meant – for most patients – that they could not physically see their relatives; however, special exceptions to the rules were made for some patients. It was unclear what indications justified circumventing the rules, and it seemed likely to depend on the individual nurses' decisions and possibly the forcefulness of patients' and relatives' arguments. The most self‐sufficient patients overcame the visitor ban by breaking the rules themselves. They might, for instance, meet with their relatives in a hallway or outside the hospital if they were able to get there:

P *Well, for me, it has been just fine, but it has… Uh, it has been a bit annoying for the family, though. I really like to get visits, right? But they take place down in the street, and I don't sit there for a long time because I become tired, umm…*

F *So just getting there so that you can meet them and coming back again, you become tired?*

P *Yes, yes, but I also like to go down there. It's just a little tricky, right? (patient 5)*

Although such meetings were described as exhausting, patients nevertheless prioritized seeing their relatives despite their fatigue.

Most patients had been able to communicate with their relatives by phone or, for some, via Skype or FaceTime video calls. As one patient described, “*We skype, talk, and email each other, and they send me pictures of what they are doing*” *(patient 10)*. This was described as the best solution for keeping in touch when nothing else was possible for talking about everyday matters and difficult issues.

However, not all patients could use these technical devices themselves and depended on the nurses to help them. For the most severely cognitively affected patients, there seemed to be another obstacle: the nurses, in some cases, confiscated the patients' telephones. The justification in these cases appeared to be that it would help the patient to remain calm or protect either the patient or their relatives since the patient might talk non‐sense or say things they would later regret. These patients become even more dependent on nurses' assistance:

R *Can you call yourself? Can you do that?*

P *No*.

R *You need help with that?*

P *Yes, I do, but I do not have a phone*.

R *You do not have a phone in here?*

P *No. The chief consultant said that it was best for me to stay calm*.

R *Okay*.

P *So, how do you talk together on FaceTime?*

R *I must ask a nurse. Then they come with the phone or iPad. (patient 11)*

It seemed that patients who could not find solutions for communicating with their relatives, such as leaving the ward or calling them by phone, depended on nurses' willingness to help and tendency to prioritize this to maintain communication. It was clear that patients experienced different circumstances.

### 4.4 The importance of compassionate nurses

Without their relatives present, the patients had a great need for compassionate nurses to meet the psychosocial needs that relatives would ordinarily have attended to. These psychosocial needs ranged from “just a little time for small talk” to “lending an ear,” “providing comfort,” and “a hand to hold.” In these scenarios, nurses were described as having the potential to make a real difference:

*I think… it's all about their personal commitment and, yes, daring to give something of themselves. They are not afraid of that. So, it's a really nice thing, and it does make things a little more human, you know, but you still miss your family. Just a smile… yes … Bloody hell, I'm looking forward to going home!* *(patient 7)*

Some patients described that the nurses could be very being busy but still pleasant and friendly:

*This coronavirus is also an extra burden for the* nurses*. Now it is not only the patients but also their relatives that they must deal with. It is quite a task…* *(patient 13)*

Another patient elaborated:

*If they had only called them (the relatives) and said I was ok. I know this is a new situation for everyone, but if they were to do it again: 'Okay, the patient is awake, then we will make a call.' I know that the nurse said they were too busy to talk on the phone all the time… But if they established calling hours, I think that would be nice for the relatives. I also believe that the nurses would then have a lot more time to take care of the patients instead of taking care of annoying relatives*. *(patient 8)*

However, the fact that patients perceived the nurses as too busy often caused the patients to refrain from asking for help to call their relatives or meet their relatives outside the ward if that was possible. Fortunately, some patients experienced that the nurses were aware of this particular need and hence took time to help patients and show empathy and compassion:

*I think the nurses on the ward manage very well… It seems like they have joined forces, and then they go the extra mile to make you feel like you are the most important person in the world, and that is quite important… knowing that you are not abandoned*. *(patient 7)*

Most of the patients experienced some communication between their relatives and the nurses. The communication could be an invitation to participate in a ward‐round by phone, receive a call after a ward‐round, or a call when a patient returned from surgery. The patients did not experience any direct involvement of relatives, for example about discharge.

## 5 DISCUSSION

Our study revealed that most patients said torn between acknowledging the need for a COVID‐19 visitor ban and attending to their suffering. All patients came to terms with the visitor ban, but this could be hard to accept whether the rules were bent for other patients. The fact that they were not allowed to bring their loved ones into the hospital was a painful experience, and some patients said that they were all alone in the world. Some patients described that the lack of contact with their relatives influenced their moods; they could easily become upset and feel as though they had lost their motivation to get better. They even described that they could lose the desire to attend to physical necessities. Thus, emotional distress directly influenced the physical course of patients' illnesses. These findings were not unexpected since relatives have been reported as essential for helping to meet patients' physical, communicative and emotional needs while keeping up hope (Loft et al., 2019). A Dutch study correspondingly illuminated how the COVID‐19 restrictions evoked feelings of fear and loneliness in outpatients in a psycho‐oncological setting, suggesting this is a cross‐sector phenomenon. These patients were often isolated in their homes and thus experienced unmet needs (Schellekens & van der Lee, 2020). The interviewed patients additionally described how the distance could complicate their relatives' involvement and cause them extra worries because neither the patients nor their relatives could see each other with their own eyes to assess how they were doing. Our findings seemed consistent with Schellekens and Van der Lee reporting that denying patients the opportunity of their relatives' presence during care has a domino effect on both patients and their families (2020). Fears and worries are transferred to the patients, who worry about being alone and dying without saying farewell to their loved ones (Schellekens & van der Lee, 2020). Being a patient with neurological or neurosurgical disease brings an extra dimension to the disease. These patients are often in vulnerable situations, with critical or acute sickness, and have challenging communicative and cognitive deficits. This vulnerability calls for systematic and deliberate actions from nurses to ensure that patients are supported in maintaining contact and relationships with their loved ones for the sake of both patients and their families. This means that nurses should support patients and take responsibility for outreach since this is an important part of nursing and not always possible for the patients themselves.

To a certain degree, many patients had been able to communicate with their relatives via phone or video calls, which was described as the best solution when nothing else was possible. Not all patients could use these technical devices themselves, and they were dependent on the nurses' willingness to prioritize and assist them in maintaining communication with their relatives. Another obstacle for the most severely affected patients was that nurses sometimes confiscated their telephones – apparently to protect them. Studies have claimed that relatives of neurological patients play a statistically significant role because the patients are often seriously ill and may suffer additional physical, cognitive, communicative, behavioural or psychosocial limitations (Ates et al., 2018; Hesselvig et al., 2020). Because of these complex circumstances, a lack of communication seems very unfortunate for both patients and their loved ones. It would be relevant to investigate the assumptions behind nurses' concerns and feelings of needing to shield patients from their loved ones. It would also be appropriate to examine the patients' and their relatives' wishes to stay in touch and how communication could best be facilitated in these situations and investigate the relatives' experiences.

Finding ways of maintaining contact despite the visitor ban was a priority for the interviewees, and it seemed important to focus on the unequal opportunities for overcoming the visitor ban. Our results illuminate a lack of clarity about the rules relating to the visitor ban and means of maintaining the contact between patients and relatives and about communication between relatives and nurses and the circumstances under which this could occur. It became evident that electronic devices were integral to keeping contact between many, including patients and their relatives. One way of supporting contact and the involvement of the relatives could be to introduce the systematic use of electronic devices into the communication between nurses, patients and relatives if, of course, this is acceptable to the patients.

Without their relatives present, the patients had a great need to meet compassionate nurses. Fortunately, some patients found that the nurses met this need and took time to help patients and express empathy and compassion. Most patients, however, did not seem to have their needs fully met in this regard. Some described that the nurses were pleasant but busy. Patients, in general, saw the nurses as extremely busy, which often caused them to refrain from asking for help. In crises such as the current pandemic, nurses may suffer high compassion fatigue and burnout levels, making it harder for them to meet their patients' emotional needs (Ruiz‐Fernández et al., 2020). We cannot rule out that this phenomenon could explain the perceived inaccessibility of nurses. Some studies have reported that the pandemic has caused severe psychological suffering in healthcare workers (Blanco‐Donoso et al., 2021; Tan et al., 2020). Ruiz‐Fernández found moderate to high compassion fatigue and burnout levels among healthcare workers during the COVID‐19 pandemic. Surprisingly, these levels appeared to be stable compared to before the pandemic and thus were not affected by the present crisis (Ruiz‐Fernández et al., 2020). Wu et al. (2020) found lower levels of burnout in frontline workers than in other healthcare professionals. Perhaps a consequence of healthcare professionals in regular wards feeling less in control and less informed about the pandemic's evolution than those working in the frontline (Wu et al., 2020). Furthermore, nurses are often perceived as task‐oriented rather than person‐centred, leaving patients’ emotional needs unmet (Belle et al., 2020). Belle et al. suggest integrating physical, psychosocial, and relational care elements as presented in the Fundamentals of Care framework for daily practice as the way forward (Belle et al., 2020), which may now be more relevant than ever. Focusing on nurses’ well‐being also seems important since nurses suffering from compassion fatigue or burnout will inevitably have difficulty meeting patients’ emotional needs.

It is worth noting that some patients said that the visitor ban gave them some peace of mind and relieved them of the responsibility to decide whether they wanted relatives to visit. In a recent similar study on relatives' experiences of the visitor ban, we found that some relatives likewise said relieved of the pressure to visit patients during the visitor ban (not yet published). It would be interesting to examine whether nurses – once the visitor ban is lifted – could play a more statistically significant role in identifying these patients and relatives and help them to reduce the number of visits when appropriate. Although little evidence exists, other studies have described some positive consequences of the COVID‐19 restrictions. Hørmann Thomsen et al. (2021) found that people with Parkinson's disease reported statistically significantly better health‐related quality of life during the COVID‐19 lockdown and argued that reduced social pressure might be part of the explanation (Hørmann Thomsen et al., 2021). Also, postpartum mothers were relieved from the pressure of daily activities during the pandemic (Joy et al., 2020). The aftermath of COVID‐19 is uncertain. Although the overall expenses are presumably high, there may be an opportunity of some positive effects. So far, published studies on this aspect of the pandemic have been carried out in its early stages. It will be interesting to monitor whether these positive effects continue after prolonged periods of restrictions.

## 6 LIMITATIONS

This study illuminates how 14 neurological patients in three different wards in Denmark experienced their hospitalization during a COVID‐19 visitor ban. Participants were chosen to facilitate wide‐range sampling and represent patients with diverse levels of disease, age and gender. Some patients had communicative disabilities or were affected by the circumstances to a degree that could have influenced the results. However, we feel that this barrier made it even more important to give these patients a voice. Additional observations could have further strengthened the study, but this was not an option due to the COVID‐19 situation. Obtaining a fuller perspective by exploring relatives' and nurses' experiences would be valuable.

## 7 CONCLUSION

Neurological patients are in a vulnerable situation when hospitalized during a COVID‐19 visitor ban. For most patients, it was a painful experience with the potential to influence their mental and physical health. Findings from our study highlight the importance of the restrictions being imposed equally on all patients in clinical practice to help neurological patients come to terms with restrictions such as a visitor ban.

Because of the complex circumstances, the lack of visitors was unfortunate for both patients and their loved ones. Maintaining communication, therefore, became a priority, but patients seemed to have unequal opportunities in this regard. This places an extra responsibility on nurses to prioritize and assist in this matter.

Without their loved ones beside them, the desolation of the patients led to a greater need for compassionate nurses. Some patients experienced empathic and compassionate care, but others perceived the nurses as too busy, which often caused them to refrain from seeking help.

Finally, it is worth noting that some patients said that the visitor ban had given them some peace of mind and relieved them of the responsibility for dealing with possibly unwanted visits from relatives. Potentially, nurses could focus on identifying these patients to help reduce the number of visits.

### RELEVANCE TO CLINICAL PRACTICE

As the COVID‐19 pandemic evolves, healthcare professionals worldwide are forced to change their clinical practices. The health crisis has not only challenged us but has facilitated new ways of delivering healthcare. We need to rapidly increase our knowledge in this area to effectively meet the healthcare needs of vulnerable patients during this change. Our healthcare systems will undoubtedly suffer economically and health‐wise from this crisis for many years to come. New ways of delivering health care might be an advantage in the long term. Increased use of technology could make healthcare more available and give timely interaction while reducing the pressure on our healthcare systems (Price et al., 2020).

### “What does this paper contribute to the wider global clinical community?”

1. This study is one of the first to explore the meaning of the changed practice under admission due to the COVID‐19 pandemic, focusing on the visitor ban some patients and relatives have experienced.
2. Understanding the patients' experience of having or not having their relatives present under their admission is essential for the quality of care and could serve as a fundament for improving future practice.

## CONFLICT OF INTEREST

The authors declared that there is no conflict of interest.

## Supporting information


Supplementary File S1

## Data Availability

Due to the possibility of participants’ privacy being compromised, the data are not publicly available. However, the data that support the findings of this study are available on request from the corre‐sponding author.
